# Supplementary material for: Investigating Owner Use of Dietary Supplements in Dogs with Canine Cognitive Dysfunction
Source: Animals (Basel). 2023 Sep 29;13(19):3056. doi: 10.3390/ani13193056 (PMC10571926; doi:10.3390/ani13193056)
Supplement: Supplementary file 1 [file animals-13-03056-s001.zip › Figure S1-Owner questionnaire Nutrition and use of dietary supplements in dogs with age related behavioral changes.pdf]

Dear patient owner, Does your elderly dog show certain behavioral changes such as confusion, disorientation, incontinence or failure to recognize familiar people? These can be harmless and part of the normal aging process, but in severe cases they can also affect the dogs' quality of life.

Various studies have already shown that diet and the use of nutritional supplements have an influence on general brain health and on the course of age-related behavioral changes.

We would like to learn more about what food or supplements you, as the owner, feed your old dog and why you chose to do so. In addition, we would like to find out if you have noticed any effect of the supplements on your dog. This will help us to treat patients with age-related behavioural changes in the future.

We are very grateful that you are interested in taking part in our survey. This will take approximately 15-20 minutes to complete.

If you have any questions or comments, please feel free to email me (julia.patricia.haake@tiho-hannover.de).

Section A: Language

A1. Choose a language:

Section B: Owner information

B1. In which country do you and your dog live?

- Abkhazia ☐
- Afghanistan ☐
- Egypt ☐
- Albania ☐
- Algeria ☐
- Andorra ☐
- Angola ☐
- Anguilla ☐
- Antarctica ☐
- America (United States) ☐
- Equatorial Guinea ☐
- Argentina ☐
- Arctic ☐
- Armenia ☐
- Aruba ☐
- Azerbaijan ☐
- Ethiopia ☐
- Australia ☐
- Azores ☐
- Bahamas ☐
- Bahrain ☐
- Bangladesh ☐
- Barbados ☐
- Belarus ☐
- Belgium ☐
- Belize ☐
- Benin ☐
- Bhutan ☐
- Bolivia ☐

B2. **Please select your gender:**

- Female ☐
- Male ☐
- Diverse ☐
- Prefer not to say ☐

B3. **Please select your age range:**

- 18-30 ☐
- 31-45 ☐
- 46-60 ☐
- 61-75 ☐
- over 75 ☐
- Prefer not to say ☐

B4. **For how many years have you owned dogs?**

Please provide an approximate number of years.

B5. **How many dogs do you currently own?**

Section C: Patient information

C1. What breed is your dog?

If your dog breed is not mentioned, please select "Other" and name it here.

If your dog is a mixed breed, please select "Mixed breed".

- Mixed breed ☐
- Airedale Terrier ☐
- Akita Inu ☐
- American Staffordshire Terrier ☐
- Australian Sheperd ☐
- Basset Hound ☐
- Beagle ☐
- Bearded Collie ☐
- Berger de Brie (Briard) ☐
- St. Bernard ☐
- Bernese Mountain Dog ☐
- Bichon Frise ☐
- Dogue de Bordeaux ☐
- Border Collie ☐
- Boston Terrier ☐
- Bouviers des Flandres ☐
- Cairn Terrier ☐
- Cavalier King Charles Spaniel ☐
- Chihuahua ☐
- Chinese Crested ☐
- Collie (long haired) ☐
- Welsh Corgi Pembroke ☐
- Dachshund ☐
- Dalmatian ☐
- German Boxer ☐
- Great Dane ☐

C2. **How old is your dog in years and months?**

If unknown, please estimate approximate age.

Years ☐☐☐☐☐☐☐☐☐☐

Months ☐☐☐☐☐☐☐☐☐☐

C3. **What is your dogs weight in kilograms?**

☐☐☐☐☐☐☐☐☐☐

C4. **What is your dogs sex?**

- male intact ☐
- male neutered ☐
- female intact ☐
- female neutered ☐

C5. **Does your dog currently have any chronic diseases? If yes, which ones?**

- None ☐
- Heart disease ☐
- Osteoarthritis ☐
- Kidney disease ☐
- Liver disease ☐
- Urinary tract disease ☐
- Diabetes mellitus ☐
- Cushing's disease ☐
- Thyroid disease ☐
- Addison's disease ☐
- Other ☐

Other

C6. **Is your dog currently on any prescribed medication?**

- Yes - Routine medications such as deworming and vaccinations ☐
- Yes - medication to support brain health ☐

No ☐

Yes - Other ☐

Yes - Other

C7. Has your dog ever had any of the following conditions?

- Urinary tract diseases ☐
- Incontinence (unwanted/involuntary passing of urine or faeces) ☐
- Thyroid diseases ☐
- Diseases of the ear/hearing ☐
- Diseases of the eye/sight ☐
- Behavioral problems ☐
- None ☐

C8. When was this disease diagnosed?

If unknown, please estimate approximate date.

If your dog has more than one disease, please state the date of the most recently diagnosed disease.

C9. How was this disease treated?

C10. Please describe your dog's behavioral problems briefly.

(Example: separation anxiety, noise phobia, unwanted hunting behavior, aggression, etc.).

C11. Is your dog currently in any pain? (e.g. joint pain, back pain, etc.)

- Yes ☐
- No ☐
- I don't know ☐
- Prefer not to say ☐

C12. Does your dog have health insurance?

- Yes ☐
- No ☐
- I don't know ☐

C13. Do you use dietary supplements yourself?

- Yes - for a specific reason ☐
- Yes - for my general health ☐
- No ☐
- Prefer not to say ☐

C14. Is your dog currently receiving dietary supplements?

- Yes - for a specific reason ☐
- Yes - for his general health ☐
- No ☐
- Prefer not to say ☐

C15. Have you noticed any changes in your dog since giving supplements?

If yes, please describe them.

- No ☐
- Yes ☐

C16. How long has your dog been receiving dietary supplements?

Years ☐

Comment

Months

Comment

Section D: Medical history

D1. Has your dog been diagnosed with cognitive dysfunction syndrome ("dementia") by a veterinarian?

- Yes
- No

D2. Which veterinarian diagnosed your dog with cognitive dysfunction syndrome?

- The family vet
- The family vet and a neurology specialist
- Other

Other

D3. Has your veterinarian performed a general examination on your dog (listening to his heart, palpation, taking temperature, etc.) and determined an underlying cause for his current symptoms?

- Yes
- No

D4. Has a veterinarian performed blood or urine tests on your dog that found an underlying cause for his current symptoms?

- Yes
- No

D5. Which of the following tests did your veterinarian perform to diagnose your dog's age-related behavioral changes?

- Blood tests
- Urine tests
- Neurological examination
- MRI scan of the brain

CT scan of the brain ☐

Cerebrospinal fluid (CSF) analysis ☐

I'm not sure which tests were performed ☐

None ☐

Other ☐

Other

D6. **What was the diagnosis for your dogs symptoms?**

Cognitive dysfunction syndrome ☐

Other ☐

Other

Section E: CADES dementia scale

Please state which behavior you have observed in your dog and how often this occurred. Assign points according to the following scheme, which will be summed up in a total score at the end.

Frequency:

Abnormal behaviour of the dog was...

0 points – never observed 2 points – observed at least once in the last 6 months 3 points – observed at least once per month 4 points – observed 2–4 times per month 5 points – observed several times a week.

E1. **Spatial orientation: How often have you observed the following behavior in your dog?**

	0 - never	2 - at least once in the last 6 months	3 - at least once per month	4 - 2 to 4 times per month	5 - several times a week
Disorientation in a familiar environment	☐	☐	☐	☐	☐
Failure to recognise familiar people and animals	☐	☐	☐	☐	☐
Abnormal response to familiar objects	☐	☐	☐	☐	☐
Aimlessly wandering (restlessness during the day)	☐	☐	☐	☐	☐
Reduced ability to do a previously learned task	☐	☐	☐	☐	☐

E2. Social interaction: Have you observed the following behavior in your dog?

	0 - never	2 - at least once in the last 6 months	3 - at least once per month	4 - 2 to 4 times per month	5 - several times a week
Changes in interaction between human and dog/dog and other dog (playing, petting)	☐	☐	☐	☐	☐
Changes in individual behaviour of the dog (exploration behaviour, play, perfomance)	☐	☐	☐	☐	☐
Response to commands and ability to learn new tasks	☐	☐	☐	☐	☐
Irritability	☐	☐	☐	☐	☐
Expression of aggression	☐	☐	☐	☐	☐

E3. Sleep-wake-cycles: Have you observed the following behavior in your dog?

	0 - never	2 - at least once in the last 6 months	3 - at least once per month	4 - 2 to 4 times per month	5 - several times a week
Abnormal behaviour during the night (wandering, vocalisation, restlessness)	☐	☐	☐	☐	☐
Switching between insomnia and excessive sleeping	☐	☐	☐	☐	☐

E4. House soiling: Have you observed the following behavior in your dog?

	0 - never	2 - at least once in the last 6 months	3 - at least once per month	4 - 2 to 4 times per month	5 - several times a week
Eliminating at home at random locations	☐	☐	☐	☐	☐
Eliminating in its kennel or sleeping area	☐	☐	☐	☐	☐
Changes in signalisation for elimination activity	☐	☐	☐	☐	☐
Eliminating indoors after a recent walk outside	☐	☐	☐	☐	☐
Eliminating at uncommon locations outside (e.g. grass, concrete)	☐	☐	☐	☐	☐

Section F: DISHAA dementia scale

Please list any behavioral abnormalities in your dog that have occurred since the age of 8 or later, or have developed or worsened since then.

Rate these abnormalities according to their severity and assign points. (Score: 0= never, 1= once a month, 2= once a week, 3= almost everyday).

F1. Disorientation: Have you observed the following behavior in your dog?

	0 - never	1 - once a month	2 - once a week	3 - almost everyday
Stares intently where there is nothing visible	☐	☐	☐	☐
Does not remember its way back home	☐	☐	☐	☐
Gets stuck behind objects or furniture	☐	☐	☐	☐
Stays on the wrong side of the door	☐	☐	☐	☐
Does not respond to certain stimuli to which it used to respond (for example, doorbell)	☐	☐	☐	☐
Does not give any signal when it wants to go out	☐	☐	☐	☐

F2. Sleep-wake-cycles: Have you observed the following behavior in your dog?

	0 - never	1 - once a month	2 - once a week	3 - almost everyday
Walks during the night (without an obvious reason), when it did not used to do this	☐	☐	☐	☐
Vocalises (barks, whines) during the night (without an obvious reason), when it did not use to do this	☐	☐	☐	☐

F3. Social interaction: Have you observed the following behavior in your dog?

	0 - never	1 - once a month	2 - once a week	3 - almost everyday
Does not recognise familiar people	☐	☐	☐	☐
Does not recognise familiar animals	☐	☐	☐	☐
Shows more signs of fear or aggression towards people and/or other dogs than it used to	☐	☐	☐	☐

F4. House soiling, learning and memory: Have you observed the following behavior in your dog?

	0 - never	1 - once a month	2 - once a week	3 - almost everyday
Urinates and/or defecates in new (inappropriate) places (when it did not use to do it)	☐	☐	☐	☐
Finds it difcult to respond to previously learned commands	☐	☐	☐	☐

F5. Activity: Have you observed the following behavior in your dog?

	0 - never	1 - once a month	2 - once a week	3 - almost everyday
Is less active or playful than it used to be	☐	☐	☐	☐
Shows repetitive behaviours (chases own tail, snaps at 'invisible' flies, etc.)	☐	☐	☐	☐
Walks without obvious purpose	☐	☐	☐	☐

F6. Anxiety: Have you observed the following behavior in your dog?

	0 - never	1 - once a month	2 - once a week	3 - almost everyday
Shows more signs of anxiety when separated from its owners than before (main signs of anxiety are shaking, shivering or trembling, excessive salivation, restlessness/agitation/pacing, whining, loss of appetite)	☐	☐	☐	☐

Section G: CCDR and C-BARQ dementia scale

G1. How often does your dog exhibit the following behavior? Please select the applicable answer for each item:

	Never	Once a month	Once a week	Once a day	More than once a day
How often does your dog pace up and down, walk in circles and/or wander with no direction or purpose?	☐	☐	☐	☐	☐
How often does your dog stare blankly at the walls or floor?	☐	☐	☐	☐	☐
How often does your dog get stuck behind objects and is unable to get around?	☐	☐	☐	☐	☐
How often does your dog fail to recognise familiar people or pets?	☐	☐	☐	☐	☐
How often does your dog walk into walls or doors?	☐	☐	☐	☐	☐
How often does your dog walk away while, or avoid, being patted?	☐	☐	☐	☐	☐

G2. Please rate your dog according to the following question:

	How often does your dog have difficulty finding food dropped on the floor?
Never	☐
1-30% of the time	☐
31-60% of the time	☐
61-99% of the time	☐
Always	☐

G3. Compared to 6 months ago, does your dog exhibit the following behaviors? Please select the correct answer for each item:

	Much less	Slightly less	The same	Slightly more	Much more
Does your dog now pace up and down, walk in circles and/or wander with no direction or purpose?	☐	☐	☐	☐	☐
Does your dog now stare blankly at the walls or floor?	☐	☐	☐	☐	☐
Does your dog urinate or defecate in an area it has previously kept clean (if your dog has never house-soiled, tick 'the same')?	☐	☐	☐	☐	☐
Does your dog have difficulty finding food dropped on the floor?	☐	☐	☐	☐	☐
Does your dog fail to recognise familiar people or pets?	☐	☐	☐	☐	☐
What is the amount of time your dog spends active now?	☐	☐	☐	☐	☐

G4. Please evaluate the following situations based on how likely your dog would respond with tense or fearful behavior.

Please select the most appropriate answer for each item:

	Never	Rarely	Sometim es	Usually	Always
When an unknown man approaches your home while you are not present.	☐	☐	☐	☐	☐
When an unknown woman approaches your home while you are not present.	☐	☐	☐	☐	☐
When an unknown child approaches your home while you are not present.	☐	☐	☐	☐	☐
When an unknown person visits you in your home.	☐	☐	☐	☐	☐

G5. Please evaluate the following situations based on how likely your dog would respond with tense or fearful behavior.

Please select the most appropriate answer for each item:

	Never	Rarely	Sometim es	Usually	Always
In response to sudden or loud noises (e.g. thunder, vacuum cleaner, car backfire, road drills, objects being dropped, etc.).	☐	☐	☐	☐	☐
With heavy traffic	☐	☐	☐	☐	☐
In response to strange or unfamiliar objects on or near the sidewalk (e.g. plastic trash bags, leaves, litter, flags flapping, etc.	☐	☐	☐	☐	☐
During a thunderstorm.	☐	☐	☐	☐	☐
When first exposed to unfamiliar situations (e.g. first car trip, first time in elevator, first visit to veterinarian, etc.)	☐	☐	☐	☐	☐
In response to wind or wind-blown moving objects.	☐	☐	☐	☐	☐

G6. How often does your dog do the following?

Please select the correct answer for each item:

	Never	Rarely	Sometimes	Usually	Always
Comes immediately when called if not on leash.	☐	☐	☐	☐	☐
Obeys immediately to the command "sit".	☐	☐	☐	☐	☐
Obey immediately to the command "Stay."	☐	☐	☐	☐	☐
Brings or tries to bring sticks, balls, etc.	☐	☐	☐	☐	☐
Seems to participate in or listen carefully to everything you do or say.	☐	☐	☐	☐	☐
Is slow to respond to an instruction.	☐	☐	☐	☐	☐
Is slow at learning new tricks or skills.	☐	☐	☐	☐	☐
Is easily distracted by interesting sights, sounds, or scents.	☐	☐	☐	☐	☐

G7. Does your dog react fearfully or insecurely to the following situation?

Please select the applicable answer for each item:

	Never	Rarely	Sometimes	Usually	Always
When the dog is examined or treated by a veterinarian.	☐	☐	☐	☐	☐
When having nails clipped by a household member.	☐	☐	☐	☐	☐
When groomed or bathed by a household member.	☐	☐	☐	☐	☐

G8. Does your dog react fearfully or insecurely to the following situation?

Please select the applicable answer for each item:

	Never	Rarely	Sometimes	Usually	Always
When approached by a similar sized or larger unknown dog.	☐	☐	☐	☐	☐
When an unknown dog of smaller size approaches.	☐	☐	☐	☐	☐

Section H: Treatment of age-related behavioral changes

H1. Has your dog EVER received any of the following medications for age-related behavioral changes (including those you are no longer using)?

- Selegiline ("Selgian", "Anipryl", "Eldepryl", "L-Deprenyl", "Zelapar"...) ☐
- Propentofylline ("Karsivan", "PropentoTab", "Canergy", "Vivitonin",...) ☐
- Diazepam ("Valium",...) ☐

Memantine ☐

Apoaequorin ("Neutricks", "Prevagen",...) ☐

None ☐

Other ☐

Other

H2. Is your dog CURRENTLY receiving any of the following medications to treat age-related behavioral changes?

Selegiline ("Selgian", "Anipryl", "Eldepryl", "L-Deprenyl", "Zelapar",...) ☐

Propentofylline ("Karsivan", "PropentoTab", "Canergy", "Vivitonin",...) ☐

Diazepam ("Valium",...) ☐

Memantine ☐

S-adenosyl-l-methionine ("SAMe" ☐

Phosphatidylserine ☐

Apoaequorin ("Neutricks", "Prevagen",...) ☐

None ☐

Other ☐

Other

H3. What dosages of medications is your dog receiving (or has received in the past) to treat the age-related behavioral changes?

Please copy the information on the bottles for all medications, including medication name, medication strength, dose, and frequency - start with the most recent medication first.

EXAMPLE - Here is how you might describe your medication:

"Selgian" 60mg tablets, 2 tablets 2x daily.

or

"Selgian" 20mg/kg daily (if the dosage is known for your dog).

Selegiline: [ ]

Propentofylline: [ ]

Other: [ ]

H4. Since when (exact date) has your dog been treated with these medications for age-related behavioral changes?

If the exact date is unknown, please estimate.

If your dog is receiving only ONE medication, please provide the date treatment was started.

If your dog is receiving MORE than one medication, please provide the date the most recent medication was added in.

[ ]

H5.

Does your dog experience any side effects from the medication against age-related behavioral changes ?

If your dog has not shown any of these side effects, please check none of these or "Not present".

Please indicate both the severity and frequency of side effects.

Please check all that apply.

	Mild	Moderate	Severe
Vomiting	☐	☐	☐
Diarrhoea	☐	☐	☐
Disorientation	☐	☐	☐
Confusion	☐	☐	☐
Aggression	☐	☐	☐
Repetitive movements	☐	☐	☐
Shaking	☐	☐	☐
Reduced appetite	☐	☐	☐
Dizziness	☐	☐	☐
Not present	☐	☐	☐

H6.

Does your dog experience any side effects from the medication against age-related behavioral changes ?

If your dog has not shown any of these side effects, please check none of these or "Not present".

Please indicate both the severity and frequency of side effects.

Please check all that apply.

	Rarely	Monthly	Weekly
Vomiting	☐	☐	☐
Diarrhoea	☐	☐	☐
Disorientation	☐	☐	☐
Confusion	☐	☐	☐
Aggression	☐	☐	☐
Repetitive movements	☐	☐	☐
Shaking	☐	☐	☐
Reduced appetite	☐	☐	☐
Dizziness	☐	☐	☐
Not present	☐	☐	☐

Section I: Diet

I1. **What type of food do you currently feed you dog?**

Please tick all that apply.

- Wet food ☐
- Dry food (complete diet) ☐
- Dry food (Mixer) ☐
- Home cooked diet ☐
- Raw food diet ☐
- Vegetarian or vegan diet ☐

Other ☐

Other

[ ]

I2. What protein and carbohydrate sources do you use in your dogs home-cooked diet? What are their proportions in the daily ration?

EXAMPLE - Here's how you might describe your dog's food composition:

Protein: chicken, 200g per day Carbohydrates: potato, 150g per day

If you add any other ingredients to your dog's ration (e.g. oils, eggs, etc.), you will have the opportunity to indicate this later.

Proteins (e.g. chicken, beef, etc.): [ ]

Carbohydrates (e.g. potato, rice, etc.): [ ]

I3. If you are currently feeding your dog a therapeutic, prescription or veterinary food, please list the name and brand:

- Hills Prescription Diet b/d dog food ☐
- Hills Prescription Diet j/d dog food ☐
- Purina Pro Plan NeuroCare dog food ☐
- I do not feed my dog therapeutic/prescription diet ☐
- Other ☐

Other

[ ]

I4. How often do you feed your dog?

- Once a day ☐
- Twice a day ☐
- Three times a day ☐
- More than three times a day ☐
- Ad libitum (free feeding, food left out) ☐

I5. How did you establish your current feeding routine?

- On the advice from my veterinarian ☐
- On the advice of the breeder/animal shelter ☐
- On the advice of another owner ☐
- Frim information on the pet food packaging ☐
- From published information in books/magazines ☐
- From published information online ☐
- Other ☐

Other

I6. Where do you usually buy your pet food?

- Supermarket ☐
- Online ☐
- Veterinary practice ☐
- Pet shop ☐
- Other ☐

Other

I7. Do you feed your dog snacks or treats?

- More than once a day ☐
- Once a day ☐
- A few times a week ☐
- A few times a month ☐
- Less than once a month ☐
- Never ☐

I8. Have you changed your dog's diet since the diagnosis of cognitive dysfunction/age-related behavioral changes?

If yes, please specify what changes were made and why you made these changes on the right.

No - My dog's diet has not been changed since the diagnosis of cognitive dysfunction ☐

Yes - I have changed my dog's diet since the diagnosis of cognitive dysfunction ☐

I9. What food were you feeding your dog prior to the diagnosis of cognitive dysfunction syndrome or age-related behavioral changes?

Please also name the product below.

Wet food ☐

Dry food (complete diet) ☐

Dry food (Mixer) ☐

Home cooked diet ☐

Raw food diet ☐

Vegetarian or vegan diet ☐

Name of product: ☐

Name of product:

I10. How would you rate your dogs physical condition?

Please tick one of the pictures below:

(The picture from WSAVA Nutrition Comittee was edited.)

Explanation:

1 - Ribs, lumbar vertebrae, pelvic bones clearly recognizable, no body fat, clearly reduced muscle mass

2 - Ribs, lumbar vertebrae, pelvic bones easily recognizable or palpable, no body fat, slightly reduced muscle mass

3 - Ribs palpable under light layer of fat, waist visible from above, abdominal line retracted

4 - Ribs difficult to palpate under thick layer of fat, no waist visible, abdominal line possibly not retracted

5 - fat deposits on rib cage, back area, base of tail, abdominal distension visible, abdominal line not retracted

- 1 ☐
- 2 ☐
- 3 ☐
- 4 ☐
- 5 ☐

I11. On average, how long do you exercise your dog per day (walks, etc.)?

- less than 1 hour ☐
- 1-3 hours ☐
- more than 3 hours ☐

I12. Does your dog have access to food sources other than your provided food and treats?

- Yes ☐
- No ☐

Section J: Use of dietary supplements

J1. Do you use dietary supplements to help manage your dogs age-related behavioral changes?

- Yes, I currently use supplements ☐
- No, I have never used supplements ☐
- No, but I used to use supplements ☐

Section K: Owners who have never used supplements in their dogs diet

K1. Please indicate why you have decided not to use dietary supplements to treat your dogs age-related behavioral changes.

- I do not believe dietary supplements have a beneficial effect on dogs with age-related behavioural changes ☐
- I was not aware that nutritional supplements could be used to manage age-related behavioural changes ☐
- There is no scientific evidence for dietary supplements having a beneficial effect on dogs with age-related behavioural changes ☐
- I am afraid that dietary supplements will harm my dog ☐
- I am uncomfortable using nutritional supplements without consulting a veterinarian ☐
- My veterinarian has advised me not to use nutritional supplements for my dog's age-related behavioural changes ☐
- The cost of supplements to treat my dog's age-related behavioural changes is too high in the long run ☐
- Other ☐

Other

K2. Would you ever start giving your dog dietary supplements to manage their age-related behavioral changes?

If yes, please indicate the factors that would lead you to start feeding your dog nutritional supplements on the right.

- No ☐
- Yes ☐

Section L: Owners who used supplements in the past

L1. Why did you decide to stop dietary supplementation as additional management for your dog's age-related behavioral changes?

- I do not believe that it had a beneficial effect ☐
- There was no scientific evidence for it having a positive effect ☐
- I was afraid it would harm my dog ☐
- I felt uncomfortable using the supplement without the advice of a veterinarian ☐
- The cost was too high in the long run ☐
- My dog refused to take the supplement ☐
- The supplement had a negative impact on my dog's health ☐
- My dog had side effects after taking the supplement ☐
- Other ☐

Other

L2. Did you discuss the use of dietary supplements with your veterinarian?

- Yes ☐
- No ☐
- Prefer not to say ☐

L3. Would you ever give your dog supplements to manage his age-related behavioral changes again?

If yes, please indicate the factors that would cause you to feed your dog nutritional supplements on the right.

- No ☐
- Yes ☐

Section M: Owners who currently use supplements in their dogs diet or have used them in the past

Note:

If you used to give your dog dietary supplements in the past but have now stopped, please complete the following questions retrospectively.

M1. Where did you first hear about nutritional supplements as additional management options for age-related behavioral changes?

- From my veterinarian ☐
- From breeder/animal shelter ☐
- From another owner (directly) ☐
- From an online support group for owners ☐
- From published information in books/magazines ☐
- From information published online (e.g. articles, papers) ☐
- From seeing the products available at a pet shop or online ☐
- Other ☐

Other

M2. Why did you decide to feed supplements to treat your dog's age-related behavioral changes?

Please expalin briefly.

M3. What combination supplements do you give your dog as additional treatment for his age-related behavioral changes?

- [ ] Aktivait
- [ ] Senilife
- [ ] Denamarin
- [ ] None
- [ ] Other

Other

M4. What nutritional supplements in the form of single supplements do you give your dog as additional treatment for his age-related behavioral changes?

How often do you use them?

	Daily	Weekly	Monthly	Less than once a month	Annually	I used to, but no longer
Alpha lipoic acid	☐	☐	☐	☐	☐	☐
Arginine	☐	☐	☐	☐	☐	☐
CBD oil (Cannabidiol)	☐	☐	☐	☐	☐	☐
Carotenoids	☐	☐	☐	☐	☐	☐
Coenzyme Q10	☐	☐	☐	☐	☐	☐
DHA or EPA (certain omega-3 fatty acids)	☐	☐	☐	☐	☐	☐
fish oil	☐	☐	☐	☐	☐	☐
Flavonoids	☐	☐	☐	☐	☐	☐
Gingko biloba extract	☐	☐	☐	☐	☐	☐
Coconut oil	☐	☐	☐	☐	☐	☐
L-carnitine	☐	☐	☐	☐	☐	☐
Milk thistle	☐	☐	☐	☐	☐	☐
MCT oil	☐	☐	☐	☐	☐	☐
N-acetylcysteine	☐	☐	☐	☐	☐	☐
Phosphatidylserine	☐	☐	☐	☐	☐	☐
Resveratrol	☐	☐	☐	☐	☐	☐
S-adenosylmethionine	☐	☐	☐	☐	☐	☐
Selenium	☐	☐	☐	☐	☐	☐
Silybin-phosphatidylcholine	☐	☐	☐	☐	☐	☐
Tryptophan	☐	☐	☐	☐	☐	☐
Vitamin C (ascorbic acid)	☐	☐	☐	☐	☐	☐
Vitamin B1 to B12	☐	☐	☐	☐	☐	☐
Vitamin E	☐	☐	☐	☐	☐	☐
Beta carotene	☐	☐	☐	☐	☐	☐
None	☐	☐	☐	☐	☐	☐

M5. If you are feeding other supplements, please list them:

M6.

What dosage of nutritional supplements is your dog currently receiving to treat his cognitive dysfunction/age-related behavioral changes?

Please copy the information on the bottles or packages of all dietary supplements, including the name of the product, strength, dosage, and frequency. Start with the most recent supplement first.

EXAMPLE - Here's how you might describe your dogs dietary supplement:Medication: MCT oil, 2x daily, 5 ml.

Supplement 1

Supplement 2

Supplement 3

Supplement 4

Supplement 5

Supplement 6

Supplement 7

M7. Where do you buy or did you usually buy your supplements? Please check all that apply:

- Supermarket ☐
- Online ☐
- Veterinary clinic ☐
- Pet store ☐
- Veterinary practitioner ☐
- I cook myself ☐

Other

Other

M8. We would like to know more about how you chose your dogs supplementation regime. Please answer the following questions and select the one that applies:

	On advice from my veterinarian	On advice from the breeder/animal shelter/animal welfare organization	On advice of another owner (directly)	On advice of an online support group for owners	From published information in books/magazines	From published information on the Internet (e.g. articles/papers)
How did you choose the supplements you give to your dog for their age-related behavioural changes?	☐	☐	☐	☐	☐	☐
How did you decide where to buy the supplements?	☐	☐	☐	☐	☐	☐

M9.

Does or has your dog had any side effects from the supplement and if so, how severe were they?
If your dog has not shown any side effects, please check "not present".

Please check ALL that apply. Please indicate the severity of the side effects. If it is useful, please also indicate the frequency of the side effects.

	Mild	Moderate	Severe
Increased appetite	☐	☐	☐
Weight gain	☐	☐	☐
Drinking more	☐	☐	☐
Drinating more	☐	☐	☐
Sleeps more than before	☐	☐	☐
Wobbly/uncoordinated when walking	☐	☐	☐
Restlessness/pacing	☐	☐	☐
Itching or skin rash	☐	☐	☐
Vomiting	☐	☐	☐
Diarrhoea	☐	☐	☐
Cough	☐	☐	☐
Not present	☐	☐	☐

M10.

Does or has your dog had any side effects from the supplement and if so, how severe were they?
If your dog has not shown any side effects, please check "not present".

Please check ALL that apply. Please indicate the severity of the side effects. If it is useful, please also indicate the frequency of the side effects.

	Rarely	Once a month	Once a week
Increased appetite	☐	☐	☐
Weight gain	☐	☐	☐
Drinking more	☐	☐	☐
Drinating more	☐	☐	☐
Sleeps more than before	☐	☐	☐
Wobbly/uncoordinated when walking	☐	☐	☐
Restlessness/pacing	☐	☐	☐
Itching or skin rash	☐	☐	☐
Vomiting	☐	☐	☐
Diarrhoea	☐	☐	☐
Cough	☐	☐	☐
Not present	☐	☐	☐

M11. Would you recommend the use of supplements to other owners of dogs with age-related behavioral changes?

If yes, which supplements would you recommend? Please indicate them on the right.

- No ☐
- I don't know ☐
- Yes ☐

Thank you for your help!

If you have questions or comments, please feel free to contact me (julia.patricia.haake@tiho-hannover.de).
